# Flexible Dry Electrode Based on a Wrinkled Surface That Uses Carbon Nanotube/Polymer Composites for Recording Electroencephalograms

**DOI:** 10.3390/ma17030668

**Published:** 2024-01-30

**Authors:** Jihyeon Oh, Kun-Woo Nam, Won-Jin Kim, Byung-Ho Kang, Sung-Hoon Park

**Affiliations:** Department of Mechanical Engineering, Soongsil University, 369 Sangdo-ro, Dongjak-gu, Seoul 06978, Republic of Korea; adad55515@soongsil.ac.kr (J.O.); kwn1522@naver.com (K.-W.N.); dnjswls0214@gmail.com (W.-J.K.); royce2080@naver.com (B.-H.K.)

**Keywords:** electroencephalogram, dry electrode, carbon nanotube, polymer composite, wrinkled surface

## Abstract

Electroencephalography (EEG) captures minute electrical signals emanating from the brain. These signals are vulnerable to interference from external noise and dynamic artifacts; hence, accurately recording such signals is challenging. Although dry electrodes are convenient, their signals are of limited quality; consequently, wet electrodes are predominantly used in EEG. Therefore, developing dry electrodes for accurately and stably recording EEG signals is crucial. In this study, we developed flexible dry electrodes using polydimethylsiloxane (PDMS)/carbon-nanotube (CNT) composites with isotropically wrinkled surfaces that effectively combine the advantages of wet and dry electrodes. Adjusting the PDMS crosslinker ratio led to good adhesion, resulting in a highly adhesive CNT/PDMS composite with a low Young’s modulus that exhibited excellent electrical and mechanical properties owing to its ability to conformally contact skin. The isotropically wrinkled surface also effectively controls dynamic artifacts during EEG signal detection and ensures accurate signal analysis. The results of this study demonstrate that dry electrodes based on flexible CNT/PDMS composites and corrugated structures can outperform wet electrodes. The introduction of such electrodes is expected to enable the accurate analysis and monitoring of EEG signals in various scenarios, including clinical trials.

## 1. Introduction

Electroencephalography (EEG) is a technique that externally measures and records changes in electrical neuronal activity in the brain. EEG primarily reflects the synaptic potentials of nerve cells in the cerebral cortex. These signals are generated by the opening of ion channels during neurotransmitter secretion, which enables sodium ions (Na^+^) to enter and potassium ions (K^+^) to leave the cell. This potential difference creates an electric field in which the changes are measured as brainwave signals. EEG is a physiological electrical signal expressed on the scalp arising from electrochemical reactions involving neurotransmitters in the human brain; therefore, it provides functional information about various brain regions. EEG signals are measured using electrodes attached to the scalp and have minute potentials that range from a few to hundreds of microvolts [1,2]. Currently, EEG is used in various fields, including clinical disease diagnosis [3,4,5,6], cognitive neuroscience [7,8,9,10,11], and artificial intelligence (e.g., brain–machine interfaces) [12,13,14,15,16,17]. However, the importance of developing reliable EEG electrodes for these diverse applications is being increasingly emphasized, owing to the reliability of EEG signals and their low signal-to-noise ratio (SNR) characteristics.

Ag/AgCl electrodes, commonly known as wet electrodes, are used to record biopotentials in most EEG studies. These wet electrodes use electrolyte gels to maintain stable skin contact; however, such gels can dehydrate over time, which reduces signal quality and potentially leads to skin irritation and allergies [18]. To address these issues and enable long-term recording, dry electrodes that do not require conductive gels have been proposed. Dry electrodes are designed for long-term EEG use and to provide user comfort. However, the primary challenge with dry electrodes lies in the air gap associated with electrode/skin contact, which prevents current flow, increases contact impedance, diminishes signal quality, and renders the recording susceptible to motion artifacts and external noise. Despite the development of various dry electrode structures, challenges remain. For example, microneedle electrodes offer low contact impedances but may cause discomfort and skin damage. Non-contact dry electrodes do not require complete skin contact, which enables long-term EEG recording, but have higher contact impedances owing to insulation layers, air gaps, and hair. In contrast, while flexible dry electrodes can overcome these challenges, they are susceptible to tearing and must be extremely thin to ensure proper skin contact, which makes handling difficult. Ongoing research has addressed these limitations by selecting appropriate materials and improving designs to enhance EEG signal reliability and quality.

Materials play crucial roles in the construction of flexible electrodes and significantly affect their performance. For example, carbon nanotubes (CNTs) are one-dimensional carbon nanomaterials [19,20] and graphene oxide (GO) is a two-dimensional carbon nanomaterial [21,22]. Both materials are highly conductive, biocompatible, and electrochemically active; consequently, they are widely used in various bioelectrodes, including electromyography (EMG), electrocardiography (ECG), EEG, and neural electrodes [23,24,25]. Polydimethylsiloxane (PDMS), a hydrophobic silicone, is widely used in biomedical applications because it is highly biocompatible, elastic, flexible, and chemically stable [26]. Combining these materials in flexible dry electrodes has the potential to enhance the quality and stability of EEG signal recordings.

This paper presents a novel flexible dry electrode designed to record biopotentials. The electrode was fabricated from a conductive composite composed of CNT and PDMS, and tunable adhesion was achieved by adjusting the PDMS cross-linker ratio. Additionally, it features a finely wrinkled surface that provides stable, low skin–electrode contact impedance. These fine wrinkles increase the contact area and friction between the electrode and skin, which enhances mechanical adhesion. Furthermore, the corrugated surface helps to reduce motion artifacts. Dry electrodes with flexible wrinkled surfaces based on these properties are essential for recording biopotentials. Our research ultimately aimed to improve EEG-signal reliability and quality.

## 2. Materials and Methods

### 2.1. Materials

Multiwalled carbon nanotubes (MWCNTs) (JENOTUNE 6A, JEIO, Incheon, Republic of Korea) were used as fillers to enhance the electrical conductivities of composite films. These one-dimensional carbon allotrope fillers have bundle lengths and single-strand diameters of 100–200 μm and 6 nm, respectively, and are more than 97.5 wt% pure. Polydimethylsiloxane (PDMS; Sylgard 184, Dow Corning, Midland, MI, USA), comprising a base and a curing agent, was sourced from the Dow Corning Corporation (Midland, MI, USA).

### 2.2. Preparing CNT/PDMS Composites and Fabricating EEG Electrodes

**Figure 1:**
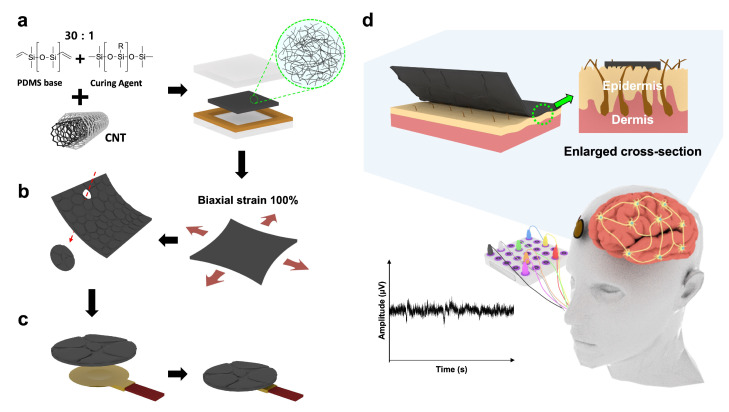
Conceptual illustration of a wrinkled CNT/PDMS electrode. (**a**) Composite preparation and degree of CNT dispersion. (**b**) Producing a wrinkled surface on the composite. (**c**) Fabricating an EEG electrode. (**d**) Fabricated EEG electrode: configuration, features, and performance.

Figure 1 outlines the key aspects of this study and shows the configuration and performance of the fabricated EEG electrodes. Henceforth, we use the term “CNTs” to refer to the multi-walled carbon nanotubes (MWCNTs) used in this study. CNT/PDMS composites were prepared with various CNT filler contents ranging from 0.5 to 10 wt%. The ratio of the PDMS base (A) to the curing agent (B) ranged between 10:1 and 30:1. The PDMS ratio limit was set to 30:1, beyond which curing was not observed. The mixture was uniformly dispersed using a paste mixer (Daehwa, Seoul, Republic of Korea) and a three-roll milling machine (Intec, Gyeonggi-do, Republic of Korea). PDMS was initially prepared by mixing A and B and then adding the CNTs to the PDMS. The paste was mixed at 500 rpm for 30 s and then continuously at 1500 rpm for 60 s. Three-roll milling was subsequently performed for 5 min to ensure appropriate dispersibility; the sample was finally cured using a hot press (Qmesys, Gyeonggi-do, Republic of Korea) at 150 °C and 15 MPa for 1 h to obtain a thin, flexible, and flat film with a thickness of 500 μm (Figure 1a). These flexible CNT/PDMS composites were used to fabricate EEG electrodes, and their flexibilities have previously been demonstrated [27,28,29].

Because the skin contracts and expands in response to minute muscle movements, we developed omnidirectionally expanding EEG electrodes that sensitively measure brain waves, even under tensile strain. Wrinkles were formed on each composite surface by 100% biaxially stretching the electrode ten times in the horizontal and vertical directions using a universal testing machine (UTM, DRTECH, Seongnam-si, Republic of Korea) (Figure 1b). The surface of the thin and flexible composite substrate undergoes physical deformation during tension and contraction to form random isotropic wrinkles [30,31,32]. Consequently, CNT/PDMS with isotropic wrinkles was obtained. A composite with a diameter of 1 cm with a wrinkled surface was fabricated using a circular punch, and the EEG electrode was finally fabricated using Ag paste (Protavic, Levallois-Perret, France) to bond the composite to the disk (Figure 1c).

### 2.3. Characterization

Surface wrinkles were investigated to evaluate the characteristics of each composite. The surface shape and three-dimensional (3D) topography of each composite was photographed using a laser confocal microscope (OLS5100, Olympus, Tokyo, Japan), and topographical roughness was quantitatively measured. Additionally, a sample was frozen in liquid nitrogen, crushed, and its shape and dispersed state were observed by field emission scanning electron microscopy (SEM; ZEISS, Baden-Württemberg, Germany) to confirm that the CNTs had been effectively dispersed in the PDMS. The instrument was operated at an accelerating voltage of 5 kV.

A multimeter (DMM7510, Keithley, Solon, OH, USA) was used in two-wire probe mode to measure the electrical conductivity and percolation threshold of each composite. Five samples (50 × 5 × 0.5 mm) with varying compositions were extracted from each composite. Ultraviolet (UV) etching was performed for 5 min using a UV–ozone chamber to reduce the contact resistance between the sample and Ag paste, which subsequently formed an electrode at each end of the sample surface. The pieces were finally cured in a convection oven at 150 °C for 1 h.

Dynamic strain testing was conducted using a custom three-dimensional stretching machine (NAMIL, Incheon, Korea) to study the effect of wrinkle formation on the composite surface. Here, strain was gradually increased from the initial state to 50%, with the change in electrical resistance continuously measured using a Keithley DMM 2110 multimeter (Keithley, Cleveland, OH, USA) and the two-wire method.

Impedances were measured to determine the intrinsic impedance (Z) of each material and the contact impedance between the skin and the electrode (Zc) across the 10–1000 Hz frequency range. A two-probe-mode LCR meter (LCR-6300, GW Instek, New Taipei, Taiwan) was used to measure impendences. Z values were measured by placing metal probes at 3 cm intervals on a prepared wrinkled CNT/PDMS sample (50 × 5 × 0.5 mm). Additionally, we fabricated CNT/PDMS samples with a diameter of 1 cm on metal plates and attached them to skin to assess Zc based on the wrinkles on the composite surface. Two sets of electrodes were placed on the arm, 2 cm apart, and a current was applied to the electrode pair to measure Zc. A 5 mV test signal was used in the 10–1000 Hz frequency range. Zc was also measured using the same method but with Ag/AgCl electrodes for comparison. Furthermore, CNT/PDMS and Ag/AgCl electrodes were attached to the arms, with fists repeatedly clenched and released to record variations attributable to motion artifacts.

A PDMS substrate was manufactured by adjusting the ratio of the PDMS crosslinker to match the Young’s modulus (140 kPa) of skin [33,34]. Afterward, a CNT/PDMS sample with each content was attached to this substrate, connected to the universal testing machine (UTM), and stretched until the composite separated. The maximum adhesion value was recorded during this process, and testing was repeated ten times. Dust adhered to the adhesive area was removed using 3M tape prior to adhesion testing. A lap-shear joint was prepared to measure the shear-adhesion strength by attaching the composite (size: 50 × 5 × 1 mm) to a PDMS substrate with an adhesive area of 1 cm^2^, after which pressure was applied using a 500 g weight for 10 s. Shear-adhesion testing was conducted at 50 mm/min and the maximum load was recorded. Shear-adhesion strength was calculated by dividing the maximum load by the initial area. Additionally, we used the pull-off method to measure tensile adhesive strength by first attaching studs to the top of a composite sample (size: 1.5 × 1.5 cm) that was then affixed to the PDMS substrate, and tensile-adhesion testing was conducted in a similar manner to that described above using a 500 g weight for 10 s. Tensile bond strength was calculated by dividing the maximum load by the initial area.

An EEG device (QEEG-64FX; Laxtha, Daejeon, Republic of Korea) was used to examine subjects that had rested for 5 min to ensure EEG stability. Electrodes were placed accurately according to the 10/20 international electrode system. This study used EEG signals in alpha-, beta-, and event-related evoked potential (ERP) tests to confirm the EEG signal-detecting ability of the developed electrodes. Alpha-band EEG signals were analyzed when subjects opened their eyes for 15 s in a relaxed state and then closed them for another 15 s. EEG signals in the beta band were analyzed when the subject solved an analytical problem. ERPs were obtained using a standard auditory oddball task (P300 acquisition) in which subjects were asked to perceive a target stimulus at 2 kHz (probability 0.2) and ignore a standard stimulus at 1 kHz (probability 0.8) [35,36]. These stimuli lasted 70 ms, included a plateau phase of 50 ms, and rise and fall times of 10 ms. Additionally, the interval between stimuli was randomly adjusted between 1.5 and 2.5 s. Subjects were instructed to close their eyes during recording to minimize electrooculography (EOG) signals. Figure 1d shows a brief schematic of the EEG recording process; the equipment used A2 as the reference electrode, with measurement electrodes attached to frontal lobe positions Fp1 and Fp2. The acquired EEG signals were amplified at a sampling rate of 1 kHz; EEG data obtained in this manner were analyzed using analysis software (TeleScan CD-TS-2.2, LAXTHA, Daejeon, Republic of Korea).

All subjects were subjected to at least five repeated trials to ensure statistical accuracy, and the resulting mean values were reported.

## 3. Results and Discussion

### 3.1. Morphologies

**Figure 2:**
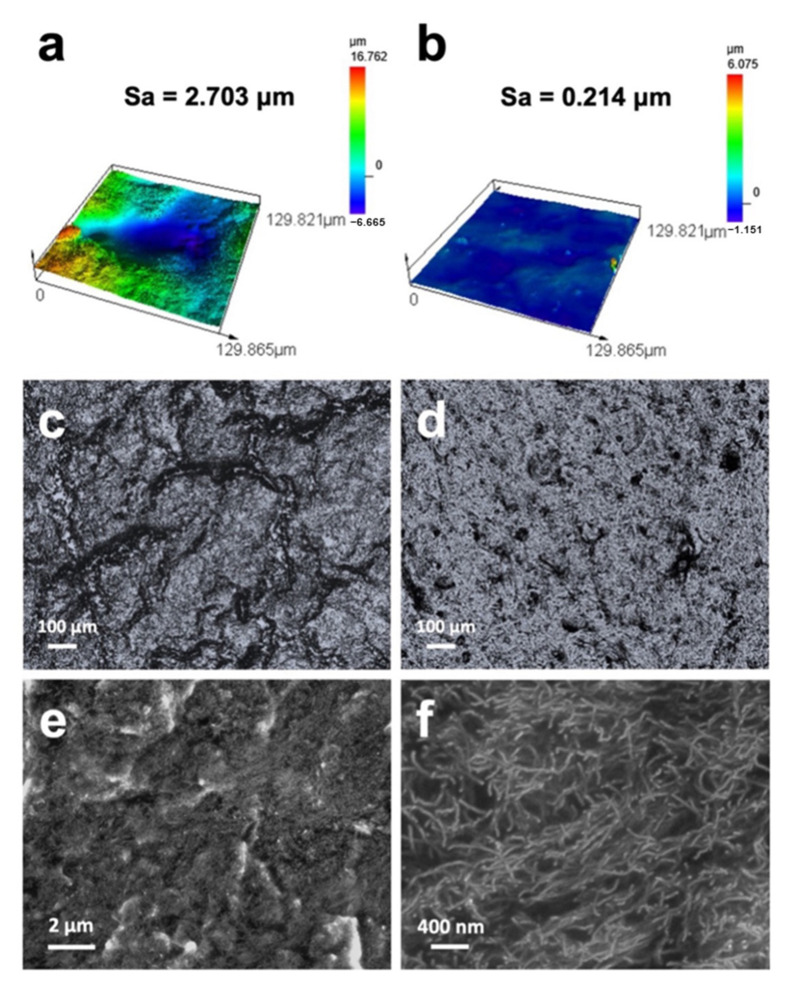
Images showing the 3D topographies and surface roughnesses of (**a**) wrinkled CNT/PDMS and (**b**) unwrinkled CNT/PDMS. Confocal images of (**c**) wrinkled CNT/PDMS and (**d**) unwrinkled CNT/PDMS. Cross-sectional SEM images of the CNT/PDMS composite at: (**e**) low resolution and (**f**) high resolution. (All images presented here are of a composite fabricated with a CNT content of 5 wt% and a PDMS A-to-B ratio of 30:1).

The surface of a fabricated composite (in this case, a composite with a CNT content of 5 wt% and a PDMS A-to-B ratio of 30:1) was examined for its topography, width, depth of texture, and height profile using a laser confocal microscope. Figure 2a,b show the three-dimensional (3D) topographies of wrinkled and unwrinkled surfaces captured at 100× magnification, along with their respective surface roughness values.

These images visually depict the wrinkles on the composite surface developed through repeated mechanical stretching. The wrinkled surface was determined to have a roughness of 2.703 μm, which is approximately 13 times that (0.214 μm) of the unwrinkled surface. Figure 2c,d show 2D images of the wrinkled and unwrinkled surfaces at 10× magnification, with the former revealing that the composite surface has randomly formed and has crumpled isotropic wrinkles with shallow divisions, in contrast to Figure 2d, which shows a smooth surface.

CNT/PDMS is endowed with the high conductivity of its CNTs and a low Young’s modulus, and effectively penetrates the valleys of curved skin, thereby maximizing the contact area and enhancing the recorded signal [37]. Therefore, CNTs must be evenly dispersed in the PDMS, and the aggregated CNTs must be well separated and electrically connected. Figure 2e,f show low- and high-resolution SEM images of the fracture surface of the fabricated composite. CNTs appear bright because they are highly conductive; consequently, the SEM images confirm that the CNTs are well separated without agglomeration. Consequently, all fillers were uniformly dispersed in the polymer matrix through the three-roll milling process.

### 3.2. Electrical Properties

#### 3.2.1. Electrical Conductivity and Percolation Threshold

**Figure 3:**
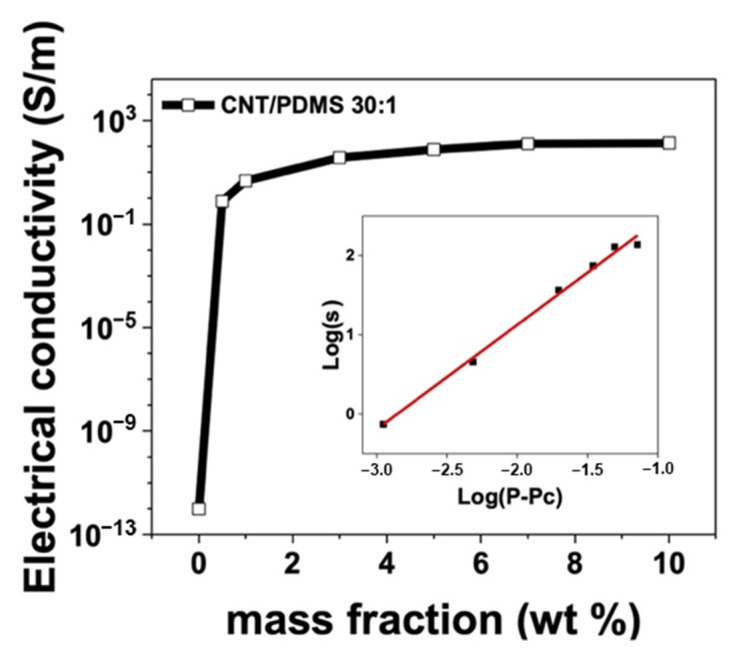
Electrical conductivity of the CNT/PDMS composite as a function of mass fraction (wt%) and percolation threshold. Inset: log-log plot of composite conductivity vs. *p* − *p_c_*.

The electrical properties of CNT/PDMS were examined by determining electrical conductivities and percolation thresholds. Figure 3 shows the electrical conductivities and percolation thresholds for CNT/PDMS with a 30:1 A-to-B ratio and various filler concentrations (0.5, 1, 3, 5, 7, and 10 wt%). Increasing the CNT content evidently enhances electrical conductivity, which primarily arises from interconnection between CNTs within the PDMS matrix, which facilitates the formation of electrical pathways. Notably, CNTs enable electrical networks to be rapidly established owing to their high aspect ratios, leading to efficient electron transport. However, the CNT volume fraction increased as its mass fraction increased, ultimately resulting in CNT saturation within the PDMS matrix. Consequently, as depicted in Figure 3, the electrical conductivity of CNT/PDMS experiences a sharp increase at low concentrations, specifically at 0.5 wt%, and becomes saturated beyond 5 wt%. The electrical conductivity of the composite was calculated using

Equation (1):

(1) $$\sigma_{conductivity}=\frac{l}{RA}$$

where $\sigma_{conductivity}$ is the electrical conductivity, *R* is the resistance, *A* is the cross-sectional area of the sample, and *l* is the distance between the two electrodes.

The change in electrical conductivity can be determined using percolation theory [38], which explains how CNTs, when present in sufficient quantities, form electrical pathways within the PDMS matrix, transforming CNT/PDMS from an insulator to a conductive material. Electrical pathways fail to start at CNT concentrations below the percolation threshold. Conversely, electrical pathways are established through a network of CNTs at CNT concentrations above the percolation threshold, which increases the electrical conductivity of the CNT/PDMS. This relationship can be quantitatively assessed using the power-law equation based on percolation theory:

(2) $$\sigma_{c}\propto \sigma_{0}{\left(p-p_{c}\right)}^{t}$$

where $\sigma_{c}$ is the electrical conductivity, *σ*_0_ is the reference electrical conductivity, *p* is the CNT content, *p_c_* is the electrical percolation threshold, and t is a critical index. The inset in Figure 3 shows the experimentally acquired curve and that fitted according to Equation (2). Both the measured and theoretically calculated electrical conductivities increase with increasing *p* − *p_c_*. Percolation threshold theory only predicts the conductivity of a composite polymer, with the actual conductivity affected by many factors, such as the polymer-matrix type, doping type, and fabrication process. Therefore, in this study, we determined the percolation threshold from the experimental data. The critical index (*t*) was calculated using OriginPro software 2022b (Origin Lab, Northampton, MA, USA) based on the percolation threshold and experimental data; the optimal value of t was determined to be 1.3 when the percolation threshold (*p_c_*) was set to 0.21 vol%, resulting in a fitting accuracy of 0.99 (chosen from a series of fitting results). The results (*p_c_* and *t*) follow the same trends as those previously reported [39].

#### 3.2.2. Evaluating Dynamic Artifacts and Measuring Impedance

**Figure 4:**
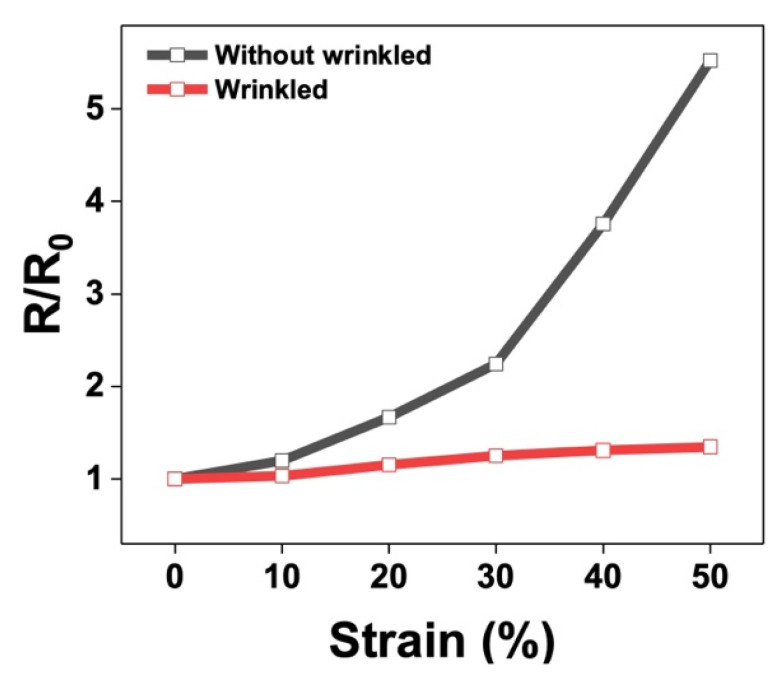
Resistances as functions of the applied uniaxial strain for wrinkled and unwrinkled electrodes.

Dynamic artifacts can detrimentally affect EEG signal quality. In this study, EEG electrodes were designed to be insensitive to dynamic deformation associated with surface wrinkling. To further investigate the influence of wrinkle geometry, the CNT content was set to 5 wt%, with the PDMS A-to-B ratio fixed at 30:1, and wrinkle-free and wrinkled samples were fabricated. The resistance of each sample was measured as it was stretched axially by up to 50%; this strain significantly exceeds the range commonly experienced by EEG electrodes. The developed electrode remained stable, even under high tensile strain. The isotropically wrinkled sample exhibited only a slight change in resistance when elongated by 50% under uniaxial strain, whereas the other sample exhibited a significant increase in R/R_0_ during elongation (Figure 4). These observations indicate that wrinkles in the direction perpendicular to stretching can facilitate the expanding shape of the electrode in a manner that absorbs the applied tensile stress and maintains nearly constant electrical resistance [40,41].

**Figure 5:**
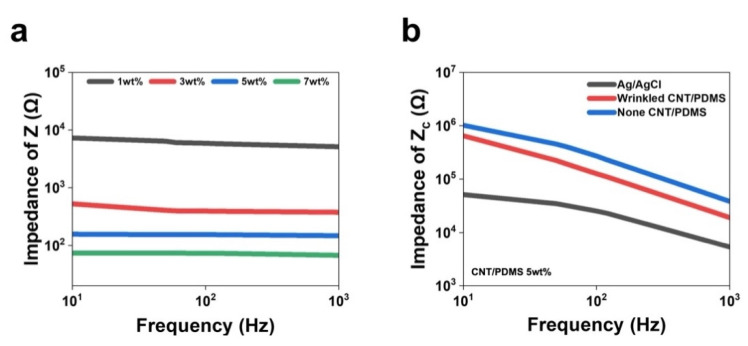
(**a**) Intrinsic impedances (Z) of wrinkled CNT/PDMS as a function of mass fraction (wt%) and frequency. (**b**) Skin contact impedances (Zc) of wrinkled and unwrinkled CNT/PDMS, and Ag/AgCl electrodes.

The electrical properties of the CNT/PDMS electrodes were investigated using the intrinsic impedance (Z) of wrinkled CNT/PDMS and contact impedance with the skin (Zc). CNT/PDMS is a mixture of CNTs and PDMS, with CNTs used to increase conductivity; hence, the CNT concentration directly determines electrical properties. The PDMS crosslinker ratio was fixed at 30:1, and electrical properties were investigated by changing the CNT mixing ratio to 1, 3, 5, and 7 wt%. Figure 5a shows that the intrinsic impedance (Z) of CNT/PDMS decreases with increasing CNT concentration, with values of 6052, 377.5, 148.2, and 67.6 Ω recorded as the CNT concentration was increased from 1 to 7 wt%, respectively. In addition, impedance did not appear to depend significantly on frequency, even as the frequency range was increased from 10 to 1000 Hz, which indicates that electrical characteristics are mainly determined by resistive components [42,43].

Skin–electrode contact impedance (Zc) is an important performance parameter for acquiring biopotentials and can depend on the skin measurement location. In this study, we conveniently measured Zc by attaching the CNT content fixed at 5 wt%, a CNT/PDMS electrode with a wrinkled surface, an unwrinkled CNT/PDMS electrode, and an Ag/Cl electrode to the forearm of a subject. Figure 5b shows that the Ag/AgCl electrode exhibited the smallest Zc value. In addition, the contact impedance of the electrode with the wrinkled surface is lower than that of the unwrinkled electrode over the entire frequency range. Specifically, the wrinkled CNT/PDMS electrode was determined to have a Zc of approximately 19 kΩ at 1 kHz, while the unwrinkled CNT/PDMS electrode exhibited a value of about 38 kΩ at the same frequency. This difference in Zc indicates that the wrinkled CNT/PDMS electrode adheres more closely to the skin. The wrinkles on the electrode surface stretch and absorb stress when tremors or movement occur, leading to the production of less noise [44,45], which supports the notion that electrodes with corrugated geometries have lower contact impedances than flat electrodes.

### 3.3. Mechanical Properties: Adhesion Testing

**Figure 6:**
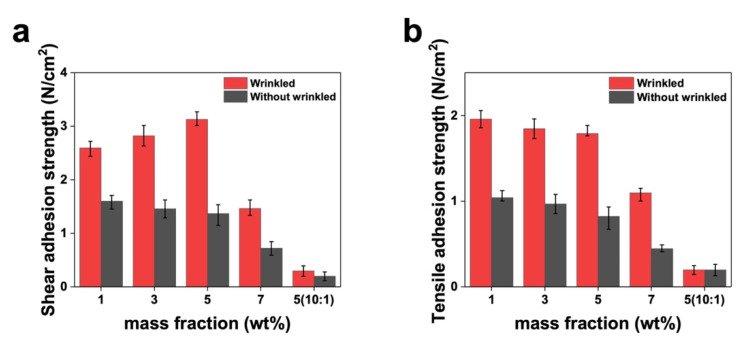
(**a**) Shear and (**b**) tensile adhesion strengths of wrinkled and unwrinkled samples as functions of mass fraction (wt%) and the PDMS cross-linker ratio in CNT/PDMS.

EEG electrode adhesion is vital for ensuring signal recording stability. In particular, adhesion quality and strength are crucial for minimizing noise caused by motion artifacts. Therefore, in this study, we measured the adhesive strengths in both shear and tensile directions by considering the forces applied to the electrodes during EEG recording. Here, we attached two types of sample, namely CNT/PDMS electrodes with wrinkles and without wrinkles, to PDMS surfaces with the same Young’s modulus as that of prefabricated skin. Attachment was achieved using a universal testing machine (UTM). The adhesive strength of each sample was quantified using ten repeated experiments. To quantify accuracy, the results of these ten measurements are represented by error bars in Figure 6. The error bars in Figure 6 were set based on the maximum and minimum values from 10 adhesion measurements. Adhesion strengths in the shear direction were measured to be 2.6, 2.8, 3.1, and 1.5 N/cm^2^ for CNT concentrations of 1, 3, 5, and 7 wt%, respectively, which confirms that adhesion strengthens as the CNT concentration is increased from 1 to 5 wt% (Figure 6a). This trend is ascribable to increased material strength with the increasing CNT concentration. As the material’s strength increases, the deformation under the same mechanical stress decreases, resulting in a higher occurrence of cracks on the surface. Consequently, a more significant number of wave-like structures (wrinkles) are formed [46]. The resulting wrinkled surface has a higher contact area, which enables the electrode to penetrate more deeply into the skin and provides strong adhesion as a consequence. Furthermore, air between the electrode wrinkles and the skin is expelled and a bond is formed with the skin when pressure is applied during the attachment of a wrinkled electrode. This process leverages the dissipation of the viscoelastic energy of the electrode, which contributes to its solid adhesion to the skin [47,48]. Applying CNT/PDMS with a corrugated surface to hairy scalp is easy, with contact remaining intact even during head movement. However, a significantly lower adhesion strength was recorded when the CNT concentration was too high (7 wt%), leading to inadequate adhesion, which is ascribable to the formation of a more significantly wrinkled pattern that impedes adhesion.

As shown in Figure 6b, tensile adhesive strength tended to decrease with increasing CNT concentration. Specifically, adhesive strength rapid declined when the concentration exceeded 5 wt%, which is attributable to inefficient skin contact owing to the presence of excessive CNT. Additionally, to assess how the PDMS crosslinker ratio affects adhesion, we prepared a 5 wt% PDMS/CNT composite with a crosslinker ratio of 10:1 and subjected it to and shear and tensile adhesion testing, which revealed a significantly lower adhesive strength compared to that recorded at a crosslinker ratio of 30:1.

The experimental results presented above confirm that CNT/PDMS-composite adhesion is closely related to the CNT concentration and Young’s modulus of the material, as well as the wrinkled nature of the surface. Figure 6 shows that unwrinkled CNT/PDMS samples have adhesion strengths that are significantly lower than that of the wrinkled CNT/PDMS samples. Improved adhesion was achieved by introducing a wrinkled electrode surface; such surfaces adhere more strongly, particularly under shear stress because the force acts in a directed parallel to the wrinkle direction, which increases friction and contributes to stronger adhesion on corrugated surfaces.

On the other hand, tensile stress acts in the vertical direction; therefore, the friction force is relatively unaffected by wrinkle shape or structure. These findings demonstrate that dry electrodes can be reused multiple times and exhibit better stability performance than wet electrodes. Improvements in adhesion were observed in various experimental scenarios. This study’s results demonstrate superior performance compared to prior research [49,50]. Based on the experimental results presented above, a CNT content of 5 wt% in 30:1 PDMS was chosen as the composition for the EEG electrode. Brain waves were subsequently recorded using electrodes manufactured with this composition.

### 3.4. Recording EEG Signals

EEG signals were recorded using a fast-Fourier-transform (FFT), event-related-potential (ERP) analysis system, with power spectra analyzed using our wrinkled flexible CNT/PDMS electrode in combination with EEG measurement software (TeleScan CD-TS-2.2, LAXTHA, Daejeon, Republic of Korea). We analyzed alpha and beta waves before and after stimulation. In this context, we defined the alpha band to range between 8 and 12 Hz and the beta band to range between 13 and 30 Hz.

**Figure 7:**
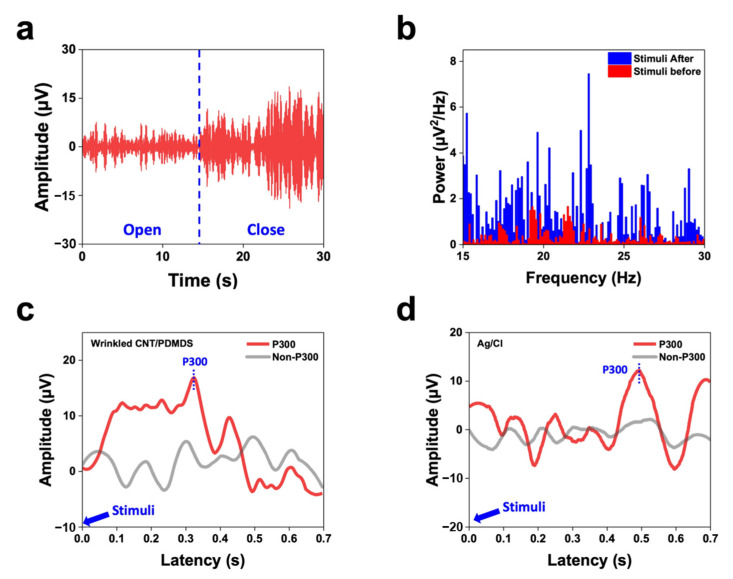
(**a**) EEG time domain recordings collected during alpha attenuation testing. Eyes were closed for 15 s. (**b**) PSD of the recorded EEG signals during rest (red bars) and problem-solving (blue bars). Auditory-evoked potentials measured using (**c**) our developed and (**d**) conventional electrodes.

We initially identified alpha band activity by observing changes in amplitude when participants alternately opened and closed their eyes, the results of which are shown in Figure 7a. Alpha waves became more intense approximately 15 s after the participants had closed their eyes, consistent with FFT alpha-band power being recorded; these signals were confirmed to correspond to alpha waves because they typically appear when the brain is relaxed. Furthermore, we examined beta band activity during rest and problem solving. The red bars in Figure 7b represent the power spectral density (PSD) at rest, whereas the blue bars represent the PSD during problem solving. Beta-band activity was observed to increase when participants engaged in specific problem-solving tasks with their eyes open and focused. The prominent blue peaks indicate increased beta-band power during stimulation.

Finally, we conducted a P300 peak-acquisition experiment, in which the brain wave response is mainly associated with auditory stimulation and temporal characteristics. This peak appeared when participants paid attention to specific stimuli or processed particular information. Figure 7c,d display the event-related potentials (ERPs) obtained using our corrugated CNT/PDMS electrodes as well as wet electrodes, which both showed the presence of P300 waves. A significant positive potential was observed between 300 and 500 ms after the onset of a “strange” stimulus, which was interpreted to be a P300 wave. The developed electrode demonstrated performance similar to that of a wet electrode and is suitable for a variety of applications. Studying P300 peaks provides essential information for neuroscience, neuropsychology, and brain–computer interface applications; hence, our electrodes are valuable tools in these fields.

## 4. Conclusions

In this study, we reliably acquired high-quality brain waves by enhancing adhesion through controlling the cross-linker ratio between CNT and PDMS, and by incorporating wrinkled structures on the composite surface. Electrodes based on a strategy that combines the low Young’s modulus and high biocompatibility of PDMS with the high conductivity of CNTs, were fabricated using CNT/PDMS composites, which was achieved by evenly dispersing agglomerated CNTs using three-roll milling technology, and confirmed by SEM. A wrinkled structure was also formed through mechanical deformation, and its suitability was confirmed using confocal imaging. The fabricated isotropically corrugated surface exhibited excellent immunity to dynamic noise, thereby enabling high-quality EEG signal recording.

Furthermore, the wrinkled CNT/PDMS electrodes effectively fill the skin layers owing to the low Young’s modulus of the composite and the nature of the wrinkled surface, which increases the contact area with the skin, in turn dramatically reducing the impedance between the skin and the electrode and significantly improving EEG signal quality. Corrugated CNT/PDMS electrodes offer the advantages of dry electrodes while maintaining low skin-contact impedances like wet electrodes, showing potential for continued use.

Moreover, reusability is an additional benefit of these corrugated CNT/PDMS electrodes, which combine the advantages of dry electrodes with highly reliable EEG measurements. The adherability of the fabricated electrodes was confirmed by measuring their shear and tensile adhesive stresses, which revealed stronger adhesion under shear stress, where the force acts in the direction parallel to the wrinkles, increasing friction and contributing significantly to superior adhesion to corrugated surfaces. EEG signals were recorded using wrinkled CNT/PDMS electrodes, which yielded excellent results in terms of power and P300 in the alpha and beta bands. Although the electrodes were attached to the forehead, significant EEG signals were recorded in response to various stimuli. These significant findings demonstrate that wrinkled CNT/PDMS electrodes provide high signal quality and can replace commercial electrodes.

## Data Availability

Data is contained within the article.
